# Partially Unroofed Coronary Sinus With Intact Atrial Septum in an Elderly Patient

**DOI:** 10.5812/cardiovascmed.4391

**Published:** 2012-11-01

**Authors:** Maryam Esmaeilzadeh, Mohammadtaghi Salehi-Omran, Saeid Hosseini, Mohammadali Sadr-Ameli

**Affiliations:** 1 Echocardiography Research Center, Rajaie Cardiovascular Medical and Research Center, Tehran University of Medical Sciences, Tehran, IR Iran; 2 Heart Valve Disease Research Center, Rajaie Cardiovascular Medical and Research Center, Tehran University of Medical Sciences, Tehran, IR Iran; 3 Cardiac Electrophysiology Research Center, Rajaie Cardiovascular Medical and Research Center, Tehran University of Medical Sciences, Tehran, IR Iran

**Keywords:** Coronary Sinus, Vena Cava, Superior, Echocardiography

## Abstract

We present a very rare case of partially unroofed coronary sinus without persistent left superior vena cava in a 74 years old female with a history of hypertension, severe myxomatous bileaflet mitral valve prolapse with severe eccentric mitral regurgitation which was diagnosed during cardiac surgery.

## 1. Introduction

Congenital anomalies of coronary sinus are rare, partly due to lack of symptoms. The most common morphological anomaly of the coronary sinus is persistent left superior vena cava which drains into the right atrium through the orifice of the coronary sinus. In these circumstances, the coronary sinus is dilated with no clinical importance. An unroofed coronary sinus, however, can produce communication into the left atrium and resulting in right-to-left shunt. The extreme form is completely unroofed coronary sinus, in which there is an interatrial communication at the mouth of the sinus. However, isolated unroofed coronary sinus can occur in the absence of persistent left superior vena cava and intact atrial septum.

## 2. Case Report

A 74 year-old female was admitted to the emergency room because of shortness of breath, palpitation and chest pain from one month ago. She had previous history of systemic hypertension and mitral valve disease and a history of hospital admission due to uncontrolled hypertension 6 months earlier. On physical examination blood pressure was considerably elevated (210/130), heart sounds were irregular with a grade IV/VI holosystolic murmur and systolic thrill in apex, lungs were clear. On ECG there was atrial fibrillation with rapid ventricular response, nonspecific ST-T changes in inferior and lateral leads. Chest-x-ray revealed mild cardiomegaly with pulmonary congestion. Transthoracic echocardiography (TTE) showed severe myxomatous bileaflet mitral valve prolapse with severe posterior directed MR jet, mild left ventricular (LV) enlargement with mild systolic dysfunction (EF = 50-55%) considering the degree of mitral regurgitation (MR), mild right ventricular (RV) enlargement with mild dysfunction , left atrial (LA) enlargement , moderate tricuspid regurgitation (TR). Pulmonary artery systolic pressure was estimated about 70 mmHg. Cardiac catheterization along with coronary angiography were done which showed significant stenosis in the first obtuse marginal branch (other coronary arteries were patent), severe mitral valve prolapse associated with severe regurgitation, severe increase in systolic pulmonary artery pressure (SPAP = 70mmHg). During cardiac surgery the anomalous connection between coronary sinus (CS) and LA was noted by the surgeon.

**Figure 1.**
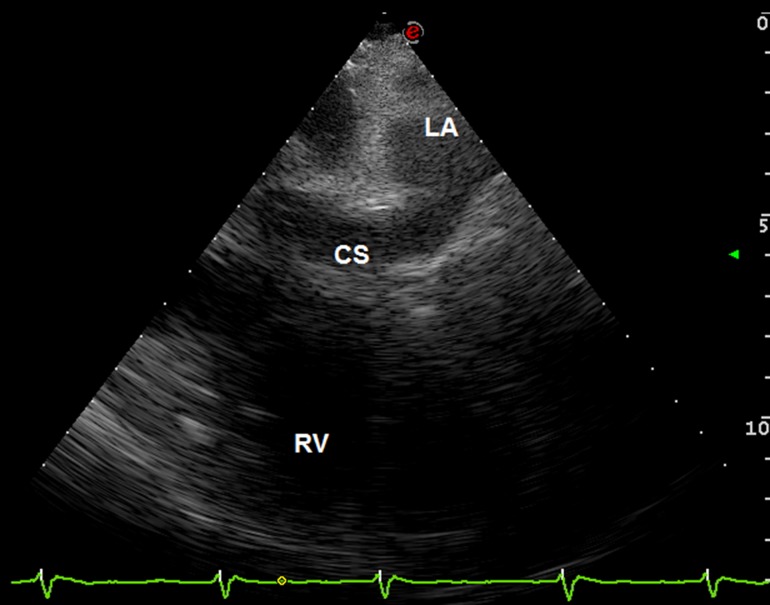
Transesophageal echocardiogram at 0 degree demonstrating the connection between the mid portion of coronary sinus and left atrium Abbreviations: CS: Coronary Sinus; LA: Left Atrium; RV: Right Ventricle

**Figure 2.**
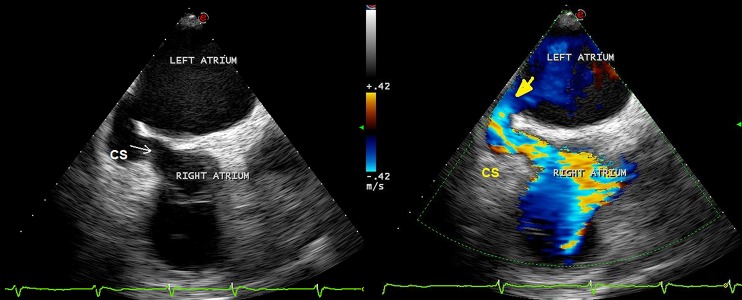
Transesophageal echocardiogram at 120 degree demonstrating the connection (yellow arrow) between the mid portion of coronary sinus and left atrium (left). Color flow Doppler revealed left to right shunt (right) Abbreviation: CS: Coronary Sinus; LA: Left Atrium

**Figure 3.**
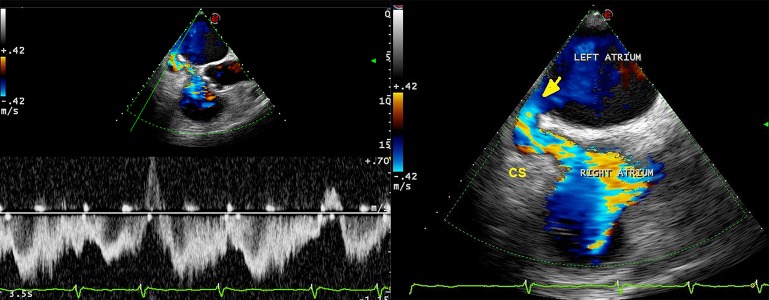
Transesophageal echocardiogram; left: Pulsed wave Doppler revealed flow through LA to CS. Right: contrast injection showed bubble passage through right atrium and CS to LA, demonstrating atrial shunt (yellow arrow) Abbreviations: CS: Coronary Sinus; LA: Left Atrium

TEE showed that in addition to its normal connection to the right atrium (RA), the CS at its mid part had a direct connection to the LA (Figure 1). Color Doppler echocardiography demonstrated left-to-right shunt (because of high LA pressure) from the LA toward CS through a moderate sized orifice (9 mm) which was located at posterosuperior portion of LA just below the right upper pulmonary vein orifice (Figure 2). The calculated left to right shunt was insignificant (QP/QS = 1.3). Agitated saline injection to left arm revealed bubble passage into the left atrium through anomalous connection between CS and LA. No persistent left superior vena cava (LSVC) was detected by CFD and contrast study (Figure3). According to the classification of Mantini et al. (1) and Edwards (2), the described malformation corresponds to the type III of unroofed coronary sinus. The post-operative course was uneventful and patient discharged after a week.

## 3. Discussion

We herein report a rare case of an isolated unroofed coronary sinus (Type III, partially unroofed mid portion) without persistent LSVC and atrial septal defect that was diagnosed as an incidental finding during cardiac surgery. Communication between the LA and CS can be misdiagnosed as ASD. CS dilation may occur in different states such as congestive heart failure (CHF), persistent SVC, and coronary artery to CS fistula. Previous classification of CS anomaly was suggested by Mantini and et al. 1 as following: “1- Enlargement of the CS with or without left-to-right shunt, 2- Absent of CS, 3- Atresia of coronary sinus ostium, 4- Hypoplastic CS”. The morphological types have been divided into four groups: type I, completely unroofed with persistent LSVC; type II, completely unroofed without persistent LSVC; type III, partially unroofed mid portion (as illustrated in our case); and type IV, partially unroofed terminal portion (3).

**Figure 4:**
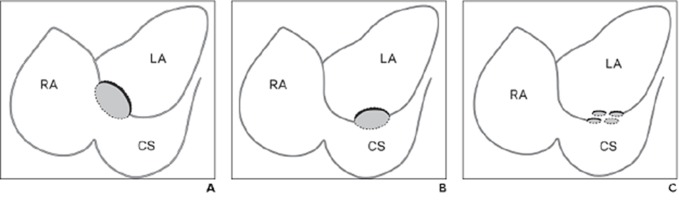
Classification of unroofed coronary sinus syndrome based on MRI and MDCT. Abbreviations: RA: Right Atrium; LA: Left Atrium; CS: Coronary Sinus A: Type 1 involves roof of CS and interatrial septum adjacent to CS orifice. B: Type 2 is defect in mid portion of roof of CS. C: Type 3 is multiple small defects in roof of CS. Adopted with permission from Kim et al. (4)

Recently Kim et.al classified unroofed coronary sinus syndrome into three types based on the morphologic features and location of the defects on cardiac magnetic resonance imaging (MRI) and multi detector CT scan (MDCT) (4). “Type 1 involves the roof of the coronary sinus and the interatrial septum adjacent to the coronary sinus orifice. Type 2 is a defect in the mid portion of the roof of the coronary sinus regardless of its size. Type 3 consists of multiple defects in the roof of the coronary sinus” (Figure 4). 

Clinical presentation and symptoms are mainly related to the size of the defect and degree of left-to-right shunt. Symptoms may vary from nonspecific or no symptoms to severe dyspnea and overt right-sided heart failure secondary to chronic RV volume overload. The diagnosis should be considered in a patient with unknown cardiac murmur, right-sided chamber enlargement, transient cyanosis or hypoxia, or paradoxical embolism like other types of atrial septal defects. Management is based on the presence of symptoms and mainly is surgical repair. Our patient was completely asymptomatic until six months ago, when she was admitted to hospital for better control of hypertension.

Although transthoracic echocardiography is the most widely used imaging technique for suspected unroofed CS but its ability to visualize the posterior structures such as the coronary sinus and pulmonary veins is limited. Transesophageal echocardiography (TEE) (5) and cardiac magnetic resonance imaging (CMR) (4, 6) are used widely to assess posterior structures precisely. Multi-detector CT has a very high spatial resolution and permits the accurate visualization and assessment of the posterior cardiac structures though (3, 4, 7). Today, with the widespread use of cardiac CT, mostly for evaluation of coronary arteries, incidental finding of asymptomatic congenital heart disease are very common. In summary, CS anomaly may occur as an isolated anomaly without clinical symptom. Although, type I and II can be determined by echocardiography in a patient who is suspected for atrial septal defect, particularly if there is considerable left to right shunt and right-sided chamber enlargement, but for diagnosis of type III (and IV) high quality CT angiography and coronary venography may be needed.
